# Expert Opinion on Benefits of Long-Chain Omega-3 Fatty Acids (DHA and EPA) in Aging and Clinical Nutrition

**DOI:** 10.3390/nu12092555

**Published:** 2020-08-24

**Authors:** Barbara Troesch, Manfred Eggersdorfer, Alessandro Laviano, Yves Rolland, A. David Smith, Ines Warnke, Arved Weimann, Philip C. Calder

**Affiliations:** 1 Nutrition Science and Advocacy, DSM Nutritional Products, 4303 Kaiseraugst, Switzerland; barbara.troesch@dsm.com (B.T.); ines.warnke@dsm.com (I.W.); 2 Department of Internal Medicine, University Medical Center Groningen, 9713 GZ Groningen, The Netherlands; dr.eggersdorfer@gmail.com; 3 Department of Translational and Precision Medicine, Sapienza University, 00185 Rome, Italy; alessandro.laviano@uniroma1.it; 4 Gérontopôle de Toulouse, Institut du Vieillissement, INSERM 1027, Centre Hospitalo-Universitaire de Toulouse, 31300 Toulouse, France; rolland.y@chu-toulouse.fr; 5 Department of Pharmacology, University of Oxford, Oxford OX1 2JD, UK; david.smith@pharm.ox.ac.uk; 6 Clinic for General, Visceral and Oncological Surgery, St. Georg gGmbH Clinic, 04129 Leipzig, Germany; Arved.Weimann@sanktgeorg.de; 7 Faculty of Medicine, University of Southampton and NIHR Southampton Biomedical Research Centre, University Hospital Southampton NHS Foundation Trust and University of Southampton, Southampton SO16 6YD, UK

**Keywords:** clinical nutrition, oral nutritional supplementation, DHA and EPA, long-chain omega-3 polyunsaturated fatty acids, inflammation, Alzheimer’s disease, immunonutrition, frailty, sarcopenia, cancer cachexia

## Abstract

Life expectancy is increasing and so is the prevalence of age-related non-communicable diseases (NCDs). Consequently, older people and patients present with multi-morbidities and more complex needs, putting significant pressure on healthcare systems. Effective nutrition interventions could be an important tool to address patient needs, improve clinical outcomes and reduce healthcare costs. Inflammation plays a central role in NCDs, so targeting it is relevant to disease prevention and treatment. The long-chain omega-3 polyunsaturated fatty acids (omega-3 LCPUFAs) docosahexaenoic acid (DHA) and eicosapentaenoic acid (EPA) are known to reduce inflammation and promote its resolution, suggesting a beneficial role in various therapeutic areas. An expert group reviewed the data on omega-3 LCPUFAs in specific patient populations and medical conditions. Evidence for benefits in cognitive health, age- and disease-related decline in muscle mass, cancer treatment, surgical patients and critical illness was identified. Use of DHA and EPA in some conditions is already included in some relevant guidelines. However, it is important to note that data on the effects of omega-3 LCPUFAs are still inconsistent in many areas (e.g., cognitive decline) due to a range of factors that vary amongst the trials performed to date; these factors include dose, timing and duration; baseline omega-3 LCPUFA status; and intake of other nutrients. Well-designed intervention studies are required to optimize the effects of DHA and EPA in specific patient populations and to develop more personalized strategies for their use.

## 1. Introduction

Life expectancy is increasing globally [1] and the prevalence of age- and lifestyle-related non-communicable diseases (NCDs), such as cancer, heart disease, respiratory disease, type 2 diabetes, obesity, chronic kidney disease and dementia is rising [2,3]. This has led patients to present with multiple co-morbidities [4,5] creating more complex needs (e.g., need for multiple medications), putting significant pressure on healthcare and social systems. Undernutrition and overnutrition can both seriously impact an individual’s risk for developing an NCD [2,3]. There is therefore a growing demand for appropriate nutrition interventions and targeted medical nutrition supplements or formulas to address patient needs, improve outcomes and help to reduce the costs of healthcare. Inflammation is considered to play a central role in age- and lifestyle-related NCDs [6], in loss of muscle mass and strength (sarcopenia) in frailty and cancer [7,8,9], and in the response to surgery and in critical illness [10]. Hence, targeting inflammation is thought to be appropriate to disease prevention and treatment. The long-chain omega-3 polyunsaturated fatty acids (LCPUFAs) docosahexaenoic acid (DHA) and eicosapentaenoic acid (EPA) are known to have roles in supporting human health [11], with one of their primary actions being to reduce inflammation [12,13,14] and promote its resolution [15,16,17]. This suggests a broad role for DHA and EPA in prevention and treatment of disease including, but not restricted to, specific therapeutic areas such as age-related decline in muscle mass, oncology, perioperative care and cognitive health.

Humans, like all mammals, cannot synthesize the essential omega-3 fatty acid α-linolenic acid. Furthermore, endogenous synthesis of EPA and DHA from α-linolenic acid is described as being poor in most humans [18] and is influenced by a range of factors such as age, sex, genetics and disease [18]. Therefore, preformed EPA and DHA must be obtained from the diet or supplements. It is now generally accepted that an intake of at least 250 mg EPA and DHA per day is required for optimal nutrition [19,20,21,22], although the exact intake required for specific populations or health conditions is not known and in many cases is likely to be in excess of this suggested minimum intake.

Blood levels of EPA and DHA are highly related to intakes [23]. Global mapping indicated low or even very low blood levels of omega-3 LCPUFAs (i.e., DHA and EPA) in a large proportion of people for whom data were available [24], suggesting low intakes in those populations. Reliance on endogenous synthesis of EPA and DHA is challenged by the low activity of this pathway [18] which is further impaired in conditions such as insulin resistance [25]. Therefore, the benefits of DHA and EPA might be particularly pronounced in those population groups with insulin resistance or other features that limit endogenous synthesis. The anti-inflammatory and inflammation resolving effects of DHA and EPA have been shown to be relevant to improved clinical outcomes in a number of specific therapeutic areas [12,13,14,15,16,17,26]. Furthermore, evidence suggests that DHA and EPA support independence in the older population, improving quality of life and significantly lowering healthcare costs [27]. Moreover, they appear to be crucial for a well-functioning immune system [28] and play an essential role in the maintenance of muscle mass and function [29], both important considerations for older people.

Adequate supply with DHA and EPA should therefore be seen as a critical component of both the prevention and treatment of many, but particularly age-related, conditions. This review aims to summarize the available evidence for DHA and EPA to promote healthy aging and to improve prognosis in a selection of medical conditions as discussed at an expert group meeting in September 2019.

## 2. The Relevance of Mechanisms of Action of DHA and EPA

**Figure 1:**
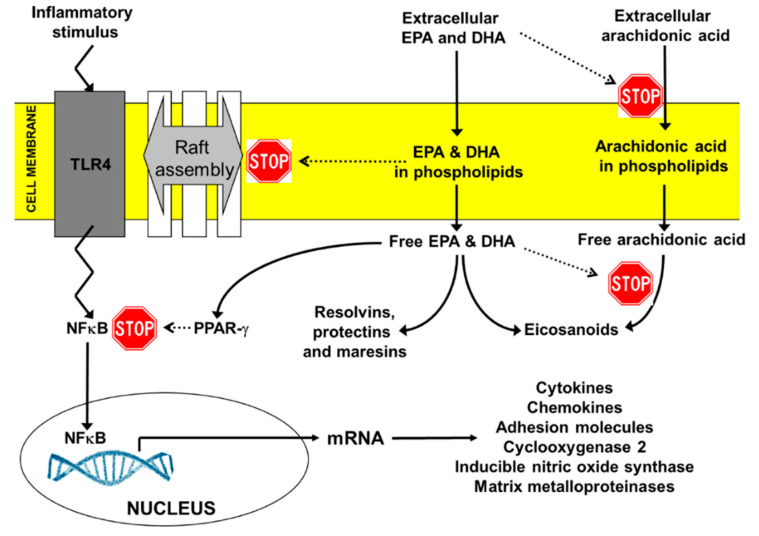
Overview of the key anti-inflammatory actions of EPA and DHA. DHA, docosahexaenoic acid; EPA, eicosapentaenoic acid; NFκB, nuclear factor kappa-light-chain-enhancer of activated B cells; PPAR, peroxisome proliferator activated receptor; TLR, toll-like receptor. Reproduced from Ref. [14].

DHA and EPA appear to act via overlapping, as well as distinct, mechanisms of action, modifying cellular function to benefit overall health and wellbeing, as well as to reduce the risk and severity of disease; these mechanisms are discussed in detail elsewhere [11,30,31]. It is their membrane-mediated mechanisms that are most well established and understood [32,33,34,35] and it is considered that through alterations at the membrane level in different cell and tissue types, DHA and EPA play an important role in cell signaling, gene expression and lipid mediator production [36]. These mechanisms are quite well explored in the context of omega-3 LCPUFA regulation of inflammatory processes, as described in detail elsewhere [12,13,14] (Figure 1). For example, increased intake of EPA and DHA results in enhanced appearance of those fatty acids in the membrane phospholipids of cells involved in inflammation (see [12,13,14] for references). This has multiple effects. Firstly, cell membranes become more fluid, affecting the behavior of several membrane proteins, including their aggregation into signaling platforms, so-called lipid rafts (see [12,13,14] for references). As a result, transmission of inflammatory signals within cells, for example from lipopolysaccharide or saturated fatty acids, becomes blunted, resulting in reduced activation of pro-inflammatory transcription factors like nuclear factor kappa-light-chain-enhancer of activated B cells (NFκB) (see [12,13,14] for references). Such transcription factors control expression of genes encoding many cytokines, chemokines, adhesion molecules, inflammatory enzymes (e.g., cyclooxygenase-2) and proteases. Thus, though these effects are initiated at the cell membrane level, omega-3 LCPUFAs can affect multiple inflammatory mediators and their anti-inflammatory actions could be wide-ranging as a result. The second effect of increased EPA and DHA in the membranes of inflammatory cells is that they partially replace the omega-6 PUFA arachidonic acid (see [12,13,14] for references). Arachidonic acid is the usual substrate for cyclooxygenase, lipoxygenase and cytochrome P450 enzymes producing eicosanoids [37,38]; these eicosanoids (e.g., prostaglandin E_2_, leukotriene B_4_) are recognized mediators of inflammation [38]. Therefore, through the EPA- and DHA-mediated decrease in arachidonic acid availability, production of these inflammatory eicosanoids is decreased (see [12,13,14] for references). The third effect of increased EPA and DHA in the membranes of inflammatory cells is that they can be released upon cellular activation. The “free” EPA and DHA can then have further actions. For example, they can act as ligands and activators for anti-inflammatory transcription factors such as peroxisome proliferator activated receptors (see [12,13,14] for references) and they can act as substrates for synthesis of eicosanoid and docosanoid lipid mediators. Eicosanoids formed from EPA such as prostaglandin E_3_ and leukotriene B_5_ often have only weak pro-inflammatory activity (see [12,13,14] for references). However, probably more importantly, both DHA and EPA are substrates for the synthesis of highly active lipid mediators important in the resolution of inflammatory processes, including resolvins, protectins and maresins [16,17]. Together, these mediators have been termed specialized pro-resolving mediators, and they have been shown in many cell culture and animal-based models to terminate inflammatory processes by decreasing cellular activation and the production of inflammatory cytokines, chemokines, adhesion molecules, proteases and enzymes (see [16,17] for references).

The foregoing discussion has emphasized the importance of the incorporation of DHA and EPA into cell membranes in order to elicit their anti-inflammatory and inflammation resolving actions. In this regard, it is important to recognize that the incorporation of DHA and EPA into the membrane phospholipids of cells involved in inflammatory responses, and into other cells and tissues such as skeletal muscle, is dose-dependently related to their intake (see [12,13,14] for references). It is possible that the membrane changes induced by low intakes of DHA and EPA are insufficient to significantly alter cell and tissue function and therefore no biological or clinical impact would be observed. Thus, the dose of DHA and EPA used in human studies is likely to be important in terms of determining the effect seen and too low a dose could result in the absence of an effect.

## 3. Omega-3 LCPUFAs and Cognitive Decline and Dementia

With the increasingly aging population, cognitive decline has become a growing public health concern: the number of persons living with dementia is expected to nearly double every 20 years [39]. Increasing evidence indicates that poor status of essential nutrients such as omega-3 LCPUFAs is associated with increased risk of cognitive decline and of developing Alzheimer’s disease [40]. DHA is a major fatty acid in membrane phospholipids in the grey matter of the brain and makes up approximately 25% of total fatty acids in the human cerebral cortex and 50% of all polyunsaturated fatty acids in the central nervous system [34,41,42,43]. Brain DHA levels decrease with adult age [44] and seem to be particularly low among Alzheimer’s patients [45]. It is conceivable that low brain DHA contributes to the decrease in cognitive functions observed with advancing age in general and to a greater degree in dementia [43,46]. The link between low omega-3 LCPUFA status and the risk of cognitive decline is supported by the observation that a higher proportion of total omega-3 LCPUFAs in the membranes of erythrocytes, considered to be a marker of both intake and status of these fatty acids, was associated with a reduced risk of developing cognitive decline in a French cohort [47]. Assessment of individuals with Alzheimer’s disease showed lower omega-3 LCPUFA intakes and plasma phosphatidylcholine levels compared to healthy controls, but the study design did not allow to draw conclusions on causality [48]. Higher DHA in plasma phosphatidylcholine was also associated with a 47% reduction in the risk of developing all-cause dementia (RR = 0.53, 95% CI 0.29–0.97; *p* = 0.04) and a 39% reduction in risk of Alzheimer’s disease (RR = 0.61, 95% CI 0.31–1.18; *p* = 0.14) in a cohort from the Framingham Heart Study [49]. The study also showed that higher dietary DHA intake was associated with a non-significantly lower risk of developing dementia in general and Alzheimer’s disease in particular (upper quartile versus lower three quartiles: RR = 0.56, 95% CI 0.23 to 1.40; *p* = 0.22 and RR 0.63, 95% CI 0.23 to 1.72; *p* = 0.37) [49]. Alzheimer patients were found to have lower DHA levels in their brains and cerebrospinal fluid compared to cognitively healthy elderly controls [50]. Fish is an important dietary source of DHA and EPA, and observational studies have assessed the association of fish consumption with cognitive health. Some of these studies show an inverse association with dementia risk [51,52,53] or a trend for such an association [54]. However, this association is not consistently seen [55,56]. A meta-analysis of observational studies showed that an additional serving of fish per week had a significant inverse association with the risk of dementia (RR = 0.95, 95% CI 0.90 to 0.99; *p* = 0.042) and Alzheimer’s disease (RR = 0.93, 95% CI 0.90 to 0.95; *p* = 0.003) [57]. Similarly, DHA intake was inversely associated with risk of dementia (RR = 0.86, 95% CI 0.76 to 0.96; *p* < 0.001) and Alzheimer’s disease (RR = 0.63, 95% CI 0.51 to 0.76; *p* < 0.001) [57]. A meta-analysis of observational studies showed a positive association of DHA intake or plasma levels with memory in adults in general [58].

The observational studies described above cannot establish a causal link and therefore intervention trials with omega-3 LCPUFAs are important to verify that these fatty acids can beneficially modify cognitive decline. Findings from such intervention trials with omega-3 LCPUFAs are not consistent [59]. However, there are relatively few trials and these differ in the dose of DHA and EPA and type of placebo used, the duration of supplementation, sample size, the severity of cognitive decline at baseline as well as the omega-3 LCPUFA status of the participants (where this was even assessed) and the cognitive outcomes/tests used. Supplementation with omega-3 LCPUFAs had a small effect on memory [60] and executive function [61] in non-demented older people. A meta-analysis of three randomized, placebo-controlled trials with omega-3 LCPUFA supplements found no effect on severity of dementia, quality of life or mental health in patients with mild or moderate Alzheimer’s disease over 6, 12 and 18 months [62]. Intake of 600 mg EPA and 625 mg DHA per day for four months showed no effect on cognition or mood in 19 individuals with Alzheimer’s disease [48,63]. However, this was a very small study and it has also been suggested that olive oil, which was used as a placebo, may have a protective effect for Alzheimer’s disease [64] and might therefore have masked the effect of the supplementation with omega-3 LCPUFAs. Similarly, an intervention comparing 200 mg EPA plus 500 mg DHA daily for 24 months compared to olive oil did not find an effect on the California Verbal Learning Test in cognitively healthy older adults (mean age 75 years) [65]. Daily supplementation with 1700 mg DHA and 600 mg EPA for six months did not affect the Mini-Mental State Examination (MMSE) score in acetylcholine esterase inhibitor treated patients with Alzheimer’s disease compared to a placebo [66]. However, the intervention had a significant effect on cognitive functioning measured with the Alzheimer’s Disease Assessment scores as well as the sub-items, and a correlation was found with the increase in plasma omega-3 LCPUFAs [67]. This suggests that the effect of omega-3 LCPUFAs depends on the specific aspect of cognitive health assessed. Moreover, subgroup analysis showed a benefit of omega-3 LCPUFAs in the group with very mild cognitive decline (MMSE score > 27) at baseline [66]. This is in line with the results from other trials indicating that interventions with DHA and EPA are less likely to have a beneficial effect on individuals experiencing dementia that has progressed beyond the mild stage [57,68,69,70,71]. A recent systematic review also reached the conclusion that the most beneficial effect of EPA and DHA supplementation in Alzheimer’s patients can be expected in the early stage of the disease [72].

While individuals with mild cognitive decline are a promising target group, it might make sense to start the intervention even earlier, in older individuals with subjective cognitive decline [73]. It has been shown that supplementation in healthy older people has a beneficial effect on white matter microstructural integrity, grey matter volume in specific brain areas and vascular parameters accompanied by improved executive function [61]. This indicates that there might be a potential for preventive uses of omega-3 LCPUFAs to maintain cognitive health in older people. However, such an effect is difficult to show as the decrease over time in the placebo group will likely be too small to show a significant difference between the groups as seen in a supplementation trial in cognitively healthy older people [74]. Therefore, careful selection of the study population is required to find the window of opportunity during which the disease has not progressed too far but is already accelerating at a sufficient speed to be able to detect a difference in the decline between the intervention and the placebo groups.

The Multidomain Alzheimer Preventive Trial (MAPT) assessed whether a multimodal intervention consisting of nutritional counseling, physical exercise and cognitive stimulation, in combination with DHA and EPA, is effective in slowing cognitive decline in older at-risk adults [75]. Three years supplementation with 800 mg DHA and 225 mg EPA showed no significant effect on cognitive decline in older people with memory complaints [76]. However, in a subgroup analysis only including individuals with low omega-3 LCPUFA status at baseline, the supplementation had a beneficial effect on cognition [77]. This indicates that people with low intakes or status of DHA and EPA should be targeted with such interventions as they may be more likely to experience the greatest benefit. Not surprisingly, the dose of DHA and EPA provided in the intervention group also plays an important role and doses below 1000 mg have not had a major effect on cognitive health in older people with some degree of cognitive decline [59].

Several trials investigating the effect of omega-3 LCPUFAs on cognitive outcomes, including decline, have been relatively short, perhaps too short to significantly affect these outcomes. It has even been suggested that the three years of supplementation evaluated in the MAPT might have been too short [78]. As neurodegeneration develops over a considerable time, longer-term intervention might be required for a benefit to manifest. A systematic review and meta-analysis of available data from animal studies suggest >10% of average total lifespan interventions had significant effects on cognitive function, neuronal loss and the amount of amyloid-beta deposits in the brain [79], but this period is considerably longer than the interventions in humans performed to date.

In addition to omega-3 LCPUFA dose, study duration and the rate of cognitive decline, other factors may also be relevant to whether an effect of these fatty acids is seen. These include the status of other nutrients and an individual’s genotype. A re-analysis of the patients assessed in the OmegAD trial [65,66] found that those with low blood homocysteine, indicating good B vitamin status, benefitted cognitively and clinically from the combined DHA and EPA treatment, whereas those with high homocysteine did not [80]. Similarly, it had been shown that those older people with mild cognitive impairment who had the highest levels of plasma omega-3 LCPUFAs benefited most from supplementation with B vitamins [81,82]. In addition, adequate intake and status of antioxidants might be required for an optimal effect of DHA and EPA on cognitive health [83].

It has been well established that apolipoprotein E (ApoE) is a very important genetic risk factor for age-dependent chronic diseases, including Alzheimer’s disease [84], but not all trials have controlled for this. Due to two major polymorphisms on the encoding exon 4 of this gene, three major protein isoforms, ApoE ε2, ApoE ε3 and ApoE ε4, exist [85]. Clinical and preclinical evidence suggests that carriers of ApoE ε4 are at a higher risk of low omega-3 LCPUFA status [86]. Moreover, it has been shown that homozygous carriers of the ApoE ε4 allele have a more than 10-fold increased risk of developing Alzheimer’s disease, possibly due to increased cholesterol levels, altered brain development early in life [84] or increased oxidative brain damage [87]. A meta-regression by Zhang et al. [57] showed that stratification by ApoE ε4 genotype had a significant effect on the association between DHA, but not EPA, intake and cognitive impairment. Another analysis found a beneficial effect of omega-3 LCPUFA supplementation on the progression of cognitive decline at an early stage in those with the ApoE ε4 genotype [59]. Thus, individuals with certain genotypes may benefit more from omega-3 LCPUFAs than those with other genotypes.

In summary, there is good evidence from observational studies for an association between DHA and slower cognitive decline or reduced risk of Alzheimer’s disease. Intervention trials are less clear, but there is some evidence that DHA and EPA can prevent or slow cognitive decline, particularly in the early stages. The inconsistent findings from trials likely relate to a number of factors including dose, duration and timing of the intervention, stage and rate of cognitive decline, status of other relevant nutrients (e.g., B vitamins) and genotype.

## 4. Omega-3 LCPUFAs and Sarcopenia and Frailty in Older People

With increasing age, achieving adequate intake of energy and essential nutrients becomes challenging due to alterations to appetite (anorexia of aging) and gastrointestinal physiology [88,89]. In addition, aging can affect dentition, gum and mouth health, and swallowing, so reducing food intake. Cognitive decline, systemic disease and use of some medications can also impact food intake. Reduced mobility, increased isolation and limited finances can restrict access to food in older people. As a consequence of these factors, malnutrition (i.e., undernutrition), frailty and sarcopenia are common and frequently overlapping conditions in older people [90,91,92]. Malnutrition is defined by ESPEN as “a state resulting from lack of intake or uptake of nutrition that leads to altered body composition (decreased fat free mass) and body cell mass leading to diminished physical and mental function and impaired clinical outcome from disease’’ [93]. Inflammation is an important contributor to the outcome of malnutrition. ESPEN recognizes disease-related malnutrition with inflammation as “a catabolic condition characterized by an inflammatory response, including anorexia and tissue breakdown, elicited by an underlying disease” [93]. Frailty is a state of vulnerability with limited reserve capacity in major organ systems; it involves weight loss, fatigue, low physical activity, slowness and weakness [94]. Frailty is associated with a higher risk of adverse outcomes such as falls, fractures, hospitalization and disability [94,95,96]. In older inpatients, frailty was found to be a risk factor for increased length of hospital stay and mortality [97,98] as well as postoperative complications [99]. Moreover, frail patients were more likely to be discharged into care homes after hospitalization [99]. A decrease in muscle mass was found to be a strong predictor of prognosis in hospitalized older people [97]. Sarcopenia is characterized by the progressive and generalized loss of skeletal muscle mass, strength and function with a consequent increased risk of adverse outcomes; the European Working Group on Sarcopenia in Older People defines sarcopenia as “a progressive and generalized skeletal muscle disorder that involves the accelerated loss of muscle mass and function” [100]. Sarcopenia is often part of the aging process preceding the onset of frailty. Age-related chronic low-grade inflammation may be an important contributor to sarcopenia [6,88,93]. Sarcopenia seems to increase the likelihood of adverse outcomes such as disability, poor quality of life and death [101,102,103]. Both muscle mass and strength were predictive for difficulties in performing activities of daily living after discharge from the hospital [104]. Sarcopenia and particularly sarcopenic obesity (i.e., low muscle mass in association with greater fat mass), have been linked to poorer prognosis, including survival, for a range of cancers [105,106,107,108,109].

Pro-inflammatory cytokines have been linked to muscle wasting [110], and consequently, the anti-inflammatory effects of omega-3 LCPUFAs may be beneficial to prevent the loss of muscle mass and strength associated with aging, sarcopenia and frailty. Furthermore, omega-3 LCPUFAs may themselves modulate muscle protein synthesis, promoting muscle strength and function [27,29], likely as a result of their incorporation into membrane phospholipids of the sarcolemma and intracellular organelles [29]. Maintenance of, or an increase in, muscle mass and function seem to be key for healthy aging [111,112], and also in recovery after surgery or during an intensive care unit (ICU) stay [113]. Long-term supplementation with DHA and EPA in older people is therefore of increasing interest as the medical community looks for safe and affordable ways to slow physical disability and improve quality of life in older individuals. Results from cross-sectional and longitudinal observational studies demonstrate that low plasma DHA and EPA levels are associated with poorer physical performance in older adults [29].

Daily supplementation with 1500 mg/d DHA and 1860 mg/d EPA for six months in healthy older men and women increased thigh muscle volume (3.6%, 95% CI 0.2% to 7.0%, *p* < 0.05), handgrip strength (2.3 kg, 95% CI 0.8 to 3.7 kg, *p* < 0.05) and one-repetition muscle strength (4.0%, 95% CI 0.8% to 7.3%, *p* < 0.05) and showed a trend towards increased average isokinetic power (5.6%, 95% CI 0.6% to 11.7%, *p* = 0.075) compared to a control group [114]. The intervention had no significant effect on body weight, total-body fat mass or the intermuscular fat content and raised no safety concerns [114]. In post-menopausal women aged > 65 years, supplementation with 720 mg/d EPA and 40 mg/d DHA for six months showed a positive effect on walking speed compared to the placebo group (3.0 ± 16% vs. −3.5 ± 14%, *p* = 0.038) [115]. Supplementation for 12 weeks with 1000 mg/d DHA and 2000 mg/d EPA in women aged 60 to 76 years resulted in a significant increase in lean body mass, increased resting metabolic rate and fat oxidation as well as decreasing time-to-get-up-and-go as a functional capacity measure [116]. However, 12 weeks supplementation with 440 mg/d DHA and 660 mg/d EPA had no effect on muscle mass or handgrip strength in community-dwelling older people (mean age 74.6 ± 8.0 years) [117]. In another study, 800 mg/d DHA and 225 mg/d EPA in combination with physical exercise, cognitive training and nutritional counseling had no effect on different measures of muscle strength in older people [118]. Based on the evidence from these trials, doses of 3000 mg/d of DHA plus EPA or more (with preferably more than 800 mg/d EPA) may be required for positive effects on physical performance in older adults [114,116] as lower doses have not had an effect [117,118]. Furthermore, the optimal ratio between DHA and EPA is not known and may differ between specific indications as different body compartments require distinct levels of omega-3 LCPUFAs (e.g., the brain is rich in DHA and poor in EPA). The scarcity of data from interventional studies [27] has prevented the development of strong recommendations on the use of omega-3 LCPUFAs in the prevention of sarcopenia so far. More randomized controlled trials, with different duration and doses, are needed to establish their effect on maintaining muscle mass in the elderly and to decrease the risk of sarcopenia and the related adverse effects on health and well-being, including the onset of frailty.

## 5. Omega-3 LCPUFAs for Nutritional Care of Cancer Patients

### 5.1. Omega-3 LCPUFAs and Cancer Cachexia

Cancer is a major public health concern and both the disease and its treatment are associated with decreased quality of life and significant economic burden due to high healthcare cost and loss of productivity. Increasing cancer incidence is due to several factors, including population growth and aging, as well as lifestyle and socio-economic factors. Various dietary behaviors are thought to be involved in the pathogenesis and progression of some cancers and they play a crucial role in tumor growth and spreading [119]. Two ways by which diet could exert effects in patients with cancer are by enhancing anticancer therapies, mitigating their side effects, and by favoring the resolution of paraneoplastic syndromes, which in turn impact outcome. Paraneoplastic syndromes are disorders triggered by an altered immune system response to new or abnormal growth of tissue. Cancer cachexia is the most frequent paraneoplastic syndrome in individuals with cancer [120]. Cachexia is a form of disease-related malnutrition with inflammation [93,121], and involves reduced appetite, altered utilization of nutrients, increased mobilization of amino acids and muscle protein turnover, loss of adipose tissue and infiltration of skeletal muscle with adipose tissue [122]. Left untreated, cachexia can progress in severity and contribute to the negative outcomes experienced by cancer patients, including mortality [123]. An international consensus of clinical experts defined cancer cachexia as “a multifactorial syndrome defined by an ongoing loss of skeletal muscle mass (with or without loss of fat mass) that cannot be fully reversed by conventional nutritional support and leads to progressive functional impairment” [124]. The importance of systemic inflammatory responses in cachexia is increasingly recognized, and it has been proposed to include this component in the definition of cancer cachexia [123,125]. Further supporting the causative role of inflammation in the pathogenesis and clinical features of cancer cachexia, it has been recently demonstrated that an elevation of the neutrophil-to-lymphocyte ratio, a simple and reliable marker of systemic inflammation, associates with greater weight loss and cachexia in patients with advanced cancer [126].

It has been proposed that current malnutrition rates in cancer patients are comparable to those >30 years ago, but they are less apparent as body mass index is often normal or even high, despite prevalence rates of cachexia and sarcopenia of 30% and 17% to 19%, respectively [122]. It is estimated that cancer cachexia affects around 50% to 80% of cancer patients and is responsible for approximately 20% of deaths in cancer patients [127,128]. Low muscle mass has a negative effect on treatment prognosis, resulting in reduced likelihood to complete at least three treatment cycles, more side effects and a lower chance of progression-free survival [129,130]. Moreover, it has a negative impact on toxicity of cancer treatment [131,132,133,134] and tumor progression during chemotherapy [133] and causes marked distress to patients and their families [135]. Still, it remains underdiagnosed and is often not treated properly as pharmacological therapies mostly fail to improve the condition significantly [136].

A review of available clinical trials showed that weight loss often starts very early in the disease progression, potentially even before the cancer itself is diagnosed [137]. The precise mechanisms are poorly understood, but chronic systemic inflammation seems to play a crucial role in most patients [123]. Inflammation is recognized as a hallmark feature of cancer development and progression [138] and targeting cancer-related inflammation at the local tumor microenvironment as well as in systemic circulation has the potential to favorably affect patient outcomes [139]. Optimal therapy should take into account the progression of the condition from pre-cachexia to cachexia and eventually refractory cachexia [140] and would ideally involve a multimodal approach including nutritional interventions targeting inflammation and reduced food intake as well as decreased physical function [126,141,142].

Given their ability to mitigate inflammation, DHA and EPA interventions in cancer patients have received increasing attention and the mechanisms are reviewed elsewhere [143,144,145,146]. There is evidence that DHA and EPA modulate the inflammatory response, measured as cytokines or C-reactive protein, and affect resting energy expenditure in cancer patients [147,148,149,150,151,152]. These findings are relevant, as increased levels of inflammation in cancer patients induce changes in pharmacokinetics of some anti-cancer drugs, resulting in slower clearance and increased treatment-related toxicities [139]. It has further been suggested that omega-3 LCPUFAs might play a role in mitigating the negative effect of disease as well as its treatment on gut health and microbiota composition [145]. In addition, observations of decreasing plasma levels indicate a depletion of EPA and DHA in cancer patients [153]. However, the effects of omega-3 LCPUFAs on nutritional status or meaningful clinical outcomes, such as quality of life, survival rates and treatment toxicity, are less well documented. Based on evidence from different systematic reviews [143,154,155,156,157], the ESPEN guidelines for nutrition in cancer patients state “in patients with advanced cancer undergoing chemotherapy and at risk of weight loss or malnourished, we suggest to use supplementation with long-chain omega-3 fatty acids or fish oil to stabilize or improve appetite, food intake, lean body mass and body weight” but the recommendation is graded as weak and the level of evidence as low [158]. A sub-group meta-analysis found a significant effect of high-protein, omega-3 LCPUFA-enriched oral nutritional supplements (ONS) when compared with isocaloric controls on body weight (+1.89 kg, 95% CI 0.51 to 3.27, *p* = 0.02) in cancer patients undergoing chemotherapy [159]. Two of the included studies reported an effect on muscle mass: supplementation with an omega-3 LCPUFA-enriched ONS (1000 mg/d DHA and 2200 mg/d EPA) resulted in a decrease in the loss of fat-free mass after three and five weeks in patients with non-small cell lung cancer (*p* = 0.02) [148], while an intervention with the same ONS resulted in a mean gain of 1.6 kg muscle mass in the intervention group versus a mean loss of 2 kg in controls (*p* = 0.01) [160]. A similar intervention resulted in an increase in skeletal muscle mass and lean body mass in cancer patients with omega-3 LCPUFA-enriched ONS (*p* = 0.0002, *p* < 0.0001, respectively), while no change was seen in these parameters in the group that received the standard ONS (*p* = 0.26, *p* = 0.19, respectively) [151]. Moreover, there are indications that supplementation with omega-3 LCPUFAs in combination with high protein might have a beneficial effect on quality of life in cancer patients [159]. Importantly, omega-3 LCPUFAs were shown to be safe and well tolerated by cancer patients [152,158].

In addition to their effect on lean mass in cancer patients, omega-3 LCPUFAs have potential use as adjuvants to cancer therapy [143]. They are thought to affect tumor activity through a range of mechanisms [144]. A review of the evidence of omega-3 LCPUFAs as an adjunct to chemotherapy found beneficial effects on tumor response to treatment, protection from therapy-related toxicity and maintenance of quality of life [145]. Further benefits of omega-3 LCPUFA supplementation might include reduction in cancer-related pain as well as a decrease in major depressive disorders, which are a frequent consequence of the stress and anxiety caused by a cancer diagnosis [161].

The lack of consensus on the definition of cachexia has led to the inclusion of patients at different stages of the condition into studies, which is expected to affect the outcomes significantly [141]. Inconsistent or negative outcomes in clinical trials, including those with omega-3 LCPUFAs, are often due to suboptimal study design regarding the selection of endpoint [137,152] or due to lack of randomization or (placebo) control group [141]. Moreover, the duration and size of the trials may have been too low in many cases to detect a relevant impact [159]. The timing of the intervention will likely also play a role, as a recent study only showed a benefit if nutritional interventions were initiated before chemotherapy started [162]. Considerable heterogeneity also exists in the pharmacological treatment as shown in a recent review that found 19 different combinations of chemotherapy used in seven studies on the effect of omega-3 LCPUFAs in cancer patients [152].

Dose selection and compliance also play an important role as shown by Fearon et al. [163] in a post-hoc analysis where there was a dose-response between reported intake of omega-3 LCPFA-enriched ONS and total (r = 0.50, *p* < 0.001) and lean body mass (r = 0.33, *p* = 0.036), as well as a correlation between plasma phospholipid EPA and change in total and lean body weight (r = 0.50, *p* < 0.001; r = 0.51, *p* = 0.001). This provides evidence that doses of 1000 mg/d DHA and 2200 mg/d EPA or even more are required for a significant effect on muscle mass. Others suggest the use of at least 2000 to 2500 mg/d DHA+EPA based on data from the available clinical trials on their use as adjuvants for chemotherapy [143,152].

It is increasingly recognized that multimodal interventions are most promising for the therapy of cancer cachexia, yet most of the clinical evidence is derived from trials using only a single therapy [141]. In a small feasibility trial, a combination of an omega-3 LCPUFA-enriched ONS (~1000 mg/d DHA and 2200 mg/d EPA), nutritional advice, 300 mg/d Celecoxib and exercise compared to standard of care resulted in a stabilization of body weight compared to weight loss in the control group [164]. The subsequent phase III study on this intervention is still ongoing [165]. Therefore, studies are needed that combine nutrition, including DHA and EPA, physical exercise as well as pharmacological interventions.

Studies highlighting cost-effectiveness might also be helpful in increasing acceptance of such interventions given the potential benefit and the low cost of omega-3 LCPUFA supplements. Due to the limited and inconclusive data available, many oncologists are yet to be convinced of the benefits that DHA and EPA have for cancer patients. Their interest in the mechanisms and possible therapies of cancer cachexia could be increased by the recent understanding that some mechanisms leading to cachexia are also involved in the process of metastasis [166]. If confirmed in clinical trials, early intervention with omega-3 LCPUFAs to prevent the development of cancer cachexia may also help to limit the spread of the tumor to distant organs. Epidemiological evidence indicates a benefit from supplementation with omega-3 LCPUFAs throughout the clinical journey of a cancer patient as higher intakes of these fatty acids in patients diagnosed with colorectal cancer were found to be associated with reduced specific mortality [167,168,169].

### 5.2. Omega-3 LCPUFAs as Components of Immunonutrition for Perioperative Care in Cancer Patients

Surgery leads to the release of stress hormones and inflammatory mediators proportional to the magnitude of the procedure, resulting in a metabolic imbalance towards increased catabolism [170,171]. While this serves to support tissue healing and the immune response, it favors the breakdown of muscle protein. This can be detrimental to the patient, especially when there is pre-existing malnutrition, sarcopenia, cachexia, obesity and myosteatosis [170] or in the presence of low-grade inflammation due to underlying conditions such as cancer or diabetes [172]. Malnutrition in surgical patients has been proposed as “a nutritional state in which nutrient intake does not match nutrient needs—due to underlying disease(s), the surgical stress response, chronic or acute inflammation, intestinal malabsorption (e.g., diarrhea) and/or patient-related factors (e.g., socio-economic status)—leading to losses in lean tissue and diminished function” [173]. Nutritional intervention can help reduce the stress of surgery, thereby preventing and treating catabolism and malnutrition [171]. This is thought to reduce the risk of complications, decrease the length of hospital stay and promote better functional recovery [170]. Considering the poor general health conditions of at-risk (e.g., many cancer) patients, nutritional conditioning (e.g., in the context of prehabilitation) may prepare individuals for an Enhanced Recovery After Surgery (ERAS) protocol [174]. Optimal timing for the introduction of nutritional therapy depends on the type of surgery and the general health status of the patient and needs further investigation [175,176,177,178,179,180,181].

Given their effect on inflammation mitigation, it is reasonable to expect a benefit of adding DHA and EPA to perioperative immunonutrition therapy. However, the evidence to support this is limited and most studies compared an ONS containing DHA and EPA combined with other immune modulating nutrients (i.e., arginine and nucleotides with or without glutamine) with regular hospital diet rather than with a standard ONS.

#### 5.2.1. Pre-Operative Immunonutrition in Cancer Patients

A recent meta-analysis focusing on patients with gastrointestinal cancer included 16 studies with 1387 patients, where the control group received either no supplements or an isonitrogenous standard ONS [182]. The preoperative administration of immunonutrition resulted in significantly decreased postoperative infectious complications in the combined studies (OR 0.52, 95% CI 0.38–0.71, *p* < 0.0001) as well as the studies with a standard ONS as a control (OR 0.49, 95% CI 0.28–0.85, *p* = 0.01). For length of hospital stay, significance was only reached in the combined studies (−1.57 days, 95% CI −2.48 to −0.66, *p* < 0.0001) but there was only a weak trend when compared to ONS (−1.06 days, 95% CI −2.76 to 0.63, *p* = 0.22). No significant effect was seen on non-infectious complications or mortality. Given their effect on post-operative morbidity and length of stay, the current ESPEN guideline for surgical patients advises that standard ONS are given pre-operatively to all malnourished cancer and other high-risk patients undergoing major abdominal surgery [171].

#### 5.2.2. Post- and Eri-Operative Immunonutrition in Cancer Patients

The evidence is somewhat stronger for benefits of postoperative than for preoperative immunonutrition [183], although the optimal timing for its introduction to patient treatment plans still needs further investigation. The ESPEN recommendation is that “peri- or at least postoperative administration of specific formulae enriched with immunonutrients should be given in malnourished patients undergoing major cancer surgery” [171]. Based on the duration of supplementation in the trials with positive outcomes, immunonutrition containing DHA and EPA as well as arginine and nucleotides should start five to seven days before surgery [171]. Similarly, the recommendations from the North American Surgical Nutrition Summit include five to seven days of pre-operative immunonutrition including omega-3 LCPUFAs, which should be continued well into the postoperative period [184]. It has even been suggested that the ideal period for pre-operative nutritional support is seven to 10 days—or longer for severely malnourished patients—in addition to postoperative nutritional support [185]. Patients who are severely compromised (e.g., due to cancer) should ideally receive preoperative nutrition support for more than 10 days [171]. Moreover, attenuation of the metabolic response to the stress of surgery through a range of measures including immunonutrition in the perioperative period is increasingly being recommended [184,186] as the combination of different elements, rather than a single one of them, is thought to produce the optimal outcome for patients [187].

While many of the trials in this area did not follow an ERAS program, adherence to such a protocol might further increase the benefits of immunonutrition. This is supported by evidence from a multicenter study in well-nourished cancer patients undergoing colorectal resection comparing peri-operative use of an ONS with immune-nutrients compared to a standard ONS as part of a more comprehensive ERAS protocol [188]. Immunonutrition including omega-3 LCPUFAs for seven days pre- and five days post-surgery was compared to a standard high caloric ONS and led to a decrease in the total number of complications, primarily due to a reduction in infectious complications (23.8% vs. 10.7%, *p* = 0.0007) [188].

It is evident that DHA and EPA play a role in perioperative immunonutrition in cancer patients, but more well-designed trials comparing standard to specialized (immunonutrition) ONS could provide clearer evidence for their use and confirm the optimal timing. A recent survey among gastrointestinal and oncologic surgeons in the U.S. showed the use of post-operative nutrition support was more common than pre-operative and the use of immune-nutrients was reported by approximately 25% of responders (versus approximately 80% use of protein-containing supplements) and lack of awareness was given as the major hurdle to a more widespread use [189].

## 6. Omega-3 LCPUFAs in the Nutritional Management of Critically Ill Patients

### 6.1. Omega-3 LCPUFAs in Sepsis

Sepsis is a severe clinical syndrome defined as “a life-threatening organ dysfunction due to a dysregulated host response to infection” [190]. In septic patients, inflammatory cytokines trigger the release of even more cytokines, culminating in a so-called cytokine storm that will in turn cause damage to cells and organs [191]. The outcome can be multi-organ failure and death. In addition to these hyperinflammatory processes, immune suppression also seems to play a role in sepsis and the balance between the two is thought to vary depending on host-, pathogen- and therapy-related factors [192,193]. The factors leading to sepsis are still incompletely understood and attempts to dampen the cytokine storm activation or consequences have failed in clinical trials [191].

A recent meta-analysis found a lower risk for mortality in 234 patients with sepsis who received omega-3 LCPUFAs, mainly intravenously, compared to control groups (OR 0.52, 95% CI 0.28 to 0.97, *p* = 0.04), while the reduction in infectious complications was only reported in one study and was not significant (OR 0.56, 95% CI 0.12 to 2.57, *p* = 0.45) and none of the studies reported cases of new onset of organ failure [194]. A complete interpretation of the findings of this meta-analysis is limited by the low number of included studies.

### 6.2. Omega-3 LCPUFAs in Acute Respiratory Distress Syndrome

Acute respiratory distress syndrome (ARDS) and multiple organ failure are important complications in patients with sepsis, resulting in prolonged ICU stays [194,195,196,197]. Specialized enteral formulations containing omega-3 LCPUFAs as well as other ingredients such as antioxidants are available for critically ill patients with ARDS or acute lung injury (ALI). However, the evidence for their effect is inconsistent. Early research demonstrated positive clinical outcomes such as improved oxygenation, fewer new organ failures, more ventilator- and ICU-free days as well as lower mortality when comparing these with high omega-6 PUFA or standard formulas [198,199,200,201]. However, subsequent research could not replicate these findings [202,203,204,205,206,207]. Consequently, the 2016 SCCM/ASPEN Guidelines for critically ill patients do not recommend the use of these specialized formulas for ARDS/ALI [208]. In contrast, the Canadian Clinical Practice Guidelines recommend that clinicians consider these specialized formulas with fish or borage oil and supplemental antioxidants for patients with ARDS/ALI [209]. The disparity between the two guidelines is likely related to differences in the studies included in the evaluation and the methods used for analyzing and interpreting the data to develop recommendations.

While a recent meta-analysis of 955 patients with ARDS or ALI showed no effect of enteral nutrition enriched with fish oil [210], after the exclusion of two studies using a bolus rather than continuous dose, there was evidence that omega-3 LCPUFA-containing formulas decreased mortality in critically ill patients including those with ARDS/ALI [211]. Moreover, a recent Cochrane review of these trials identified a significant improvement in blood oxygenation and significant reductions in ventilation requirement, new organ failures, length of stay in the ICU and mortality at 28 days when omega-3 LCPUFAs were used in patients with ARDS or ALI, although all-cause mortality was not significantly affected [212]. These findings are important in the context of the current coronavirus pandemic since severe COVID-19 results in ARDS and there are suggestions that omega-3 LCPUFAs could be a viable treatment that is worth investigating [213,214].

### 6.3. Omega-3 LCPUFAs in Critically Ill Surgical Patients

For critically ill surgical patients who require parenteral nutrition, intravenous lipid emulsions containing omega-3 LCPUFAs are considered safe, but parenteral nutrition should only be considered in patients who cannot be adequately enterally fed [171]. International consensus exists that a dose of 0.1 to 0.2 g/kg/d of fish oil would be appropriate for patients who require parenteral nutrition [215,216,217,218]. A recent meta-analysis of 49 prospective randomized trials showed significant benefits for the fish oil containing parenteral nutrition compared to a standard lipid emulsion [219]. The risk for infection was lowered by 40% (24 studies: RR 0.60. 95% CI 0.49 to 0.72; *p* < 0.00001). Mean length of stay in the ICU was significantly shortened (10 studies: 1.95 days; 95% CI −0.42 to −3.49; *p* = 0.01) as was the length of hospital stay (26 studies: 2.14 days, 95% CI −1.36 to −2.93; *p* < 0.00001). The risk for developing sepsis was also significantly diminished by 56% (nine studies: RR 0.44, 95%CI 0.28 to 0.70, *p* = 0.0004). Mortality was lower with 16%, but the difference did not reach significance (20 studies: RR 0.84, 95% CI 0.65 to 1.07; *p* = 0.15) [216]. Moreover, fish oil was found to be more cost-effective than parenteral nutrition with a standard intravenous lipid emulsion [220].

## 7. Discussion and Outlook

The evidence to date indicates that the provision of DHA and EPA through capsules, oral nutrition supplements, or enteral or parenteral formulas can help to regulate the inflammatory environment in a number of medical conditions and that this is linked in many cases to improved function, clinical course and outcomes. As dysregulated inflammation is a component of many acute and chronic diseases [221], the potential application of DHA and EPA is broad in terms of prevention and treatment. There is good evidence that DHA and EPA are a safe and cost-effective treatment that could benefit multiple patient outcomes. Use of DHA and EPA in some conditions is supported by their inclusion in relevant guidelines [123,158,171,184,209], although the level of evidence has sometimes been considered to be low. This is because of inconsistent data on the effect of DHA and EPA on clinical outcomes, especially in some settings. This inconsistency has limited stronger support through guidelines and has hindered the wider acceptance of the benefits of DHA and EPA in the medical community. If omega-3 LCPUFAs are effective in disease prevention and in patient care, it is important to understand the reasons behind the inconsistent findings of studies and use this information to design and conduct better clinical trials to determine if poor results may be due to a real lack of effect or to other factors. Undoubtedly the dose of DHA and EPA used is an important factor, but this is not the sole explanation for inconsistencies. Other considerations include the timing and duration of supply of DHA and EPA, EPA to DHA ratio, baseline EPA and DHA status, intake of other nutrients including omega-6 fatty acids, B vitamins and antioxidants, clinical state, and medication use. More well-designed intervention studies are required to address the relevance of these different variables in order to properly identify the effects of DHA and EPA in specific target patient populations. Such studies may lead to more personalized approaches to the provision of DHA and EPA to achieve the maximal clinical benefit. A focus on personalized approaches and knowledge of a patient’s specific nutritional and medical needs will be important to determine the route to optimal use of omega-3 LCPUFAs. This should take into account the interaction between genetics and nutrients [222] as well as the interaction among the nutrients themselves. Overall, the entirety of the evidence supports use of DHA and EPA in a range of medical conditions. Additional and good quality studies building on the experience of existing studies will strengthen the evidence base required to inform relevant guidelines in the future.
